# Effect of fibronectin, FGF-2, and BMP4 in the stemness maintenance of BMSCs and the metabolic and proteomic cues involved

**DOI:** 10.1186/s13287-021-02227-7

**Published:** 2021-03-06

**Authors:** Lingling Chen, Morgan Carlton, Xiaodan Chen, Navdeep Kaur, Hollie Ryan, Tony J Parker, Zhengmei Lin, Yin Xiao, Yinghong Zhou

**Affiliations:** 1 Hospital of Stomatology, Guangdong Provincial Key Laboratory of Stomatology & Guanghua School of Stomatology, Sun Yat-sen University, Guangzhou, 510055 Guangdong China; 2 Faculty of Health, Queensland University of Technology (QUT), Brisbane, Queensland 4000 Australia; 3 Faculty of Engineering, Queensland University of Technology (QUT), Brisbane, Queensland 4000 Australia

**Keywords:** Mesenchymal stem cells (MSCs), Growth factor, Stemness maintenance, Metabolite, Proteome, Regenerative medicine

## Abstract

**Background:**

Growing evidence suggests that the pluripotent state of mesenchymal stem cells (MSCs) relies on specific local microenvironmental cues such as adhesion molecules and growth factors. Fibronectin (FN), fibroblast growth factor 2 (FGF2), and bone morphogenetic protein 4 (BMP4) are the key players in the regulation of stemness and lineage commitment of MSCs. Therefore, this study was designed to investigate the pluripotency and multilineage differentiation of bone marrow-derived MSCs (BMSCs) with the introduction of FN, FGF-2, and BMP4 and to identify the metabolic and proteomic cues involved in stemness maintenance.

**Methods:**

To elucidate the stemness of BMSCs when treated with FN, FGF-2, and BMP4, the pluripotency markers of OCT4, SOX2, and c-MYC in BMSCs were monitored by real-time PCR and/or western blot. The nuclear translocation of OCT4, SOX2, and c-MYC was investigated by immunofluorescence staining. Multilineage differentiation of the treated BMSCs was determined by relevant differentiation markers. To identify the molecular signatures of BMSC stemness, gas chromatography-mass spectrometry (GC-MS), liquid chromatography-tandem mass spectrometry (LC-MS/MS), and bioinformatics analysis were utilized to determine the metabolite and protein profiles associated with stem cell maintenance.

**Results:**

Our results demonstrated that the expression of stemness markers decreased with BMSC passaging, and the manipulation of the microenvironment with fibronectin and growth factors (FGF2 and BMP4) can significantly improve BMSC stemness. Of note, we revealed 7 differentially expressed metabolites, the target genes of these metabolites may have important implications in the maintenance of BMSCs through their effects on metabolic activity, energy production, and potentially protein production. We also identified 21 differentially abundant proteins, which involved in multiple pathways, including metabolic, autophagy-related, and signaling pathways regulating the pluripotency of stem cells. Additionally, bioinformatics analysis comfirned the correlation between metabolic and proteomic profiling, suggesting that the importance of metabolism and proteome networks and their reciprocal communication in the preservation of stemness.

**Conclusions:**

These results indicate that the culture environment supplemented with the culture cocktail (FN, FGF2, and BMP4) plays an essential role in shaping the pluripotent state of BMSCs. Both the metabolism and proteome networks are involved in this process and the modulation of cell-fate decision making. All these findings may contribute to the application of MSCs for regenerative medicine.

**Supplementary Information:**

The online version contains supplementary material available at 10.1186/s13287-021-02227-7.

## Introduction

Mesenchymal stem cells (MSCs) are being exploited as an experimental therapy for many diseases in view of their self-renewable capacity and the potential to differentiate into various mesodermal lineages [1–3]. Under certain pathological conditions, the homing, recruitment, and transplantation of MSCs could help tissue repair and regeneration, leading to the recovery of injuries and degeneration disorders, making MSCs promising candidates for cell-based therapies [3–5]. However, the study of MSC populations leads to the observation that in vitro expansion of these cells is coupled with a limited cell population doubling. That is, MSCs tend to senesce and lose their multi-potentiality and self-renewal capability with time in the current cell culture method [6–8]. Therefore, creating a favorable microenvironment for maintaining the pluripotency of MSCs is of significant importance.

Growing evidence suggests that the cell fate decision of MSCs relies on genes orchestrating cell stemness or commitment to specific lineages [7, 9–11]. Recent progress in our understanding of the transcriptional regulatory circuitry in regulating the pluripotency and self-renewal of stem cells underlines the importance of transcription factors such as OCT4, SOX2, and c-MYC, which are considered to be responsible for pluripotency and self-renewal of stem cells [11–15]. Multiple levels of cell-autonomous and extrinsic signals tightly control fate determination of MSCs [11, 16–19]. It is possible that extrinsic factors derived from the local niche and systemic environment shape the epigenetic landscape of stem cells, which influences the gene expressions of stemness markers to dictate cell fate [20–26]. Previous studies revealed that designing artificial niches through adding extracellular matrix (ECM) proteins or growth factors in the ex vivo culture condition proved to be an effective way to maintain the potential capability of BMSCs [7, 26–28]. Fibronectin (FN) is a promising extracellular matrix factor for the maintenance of stemness characteristics due to its Arg-Gly-Asp containing central cell-binding domain that is essential to mesenchymal cell survival and growth [29]. Fibroblast growth factor 2 (FGF2) is a prerequisite for stem cell multi-potency since its absence triggers lineage differentiation [26, 27]. Bone morphogenetic protein-4 (BMP4) has been recognized as an extracellular pluripotency cue, and it has distinct effects on the fate determination of stem cells by shielding them from differentiation through interaction with a specific niche [30]. Interestingly, the effect of FN, FGF2, or BMP4 on self-renewal capabilities is frequently associated with transcriptional factors (OCT4, SOX2, and c-MYC) [31–34].

Therefore, based on the above, this study aimed at investigating the expression pattern of OCT4, SOX2, and c-MYC in MSCs with successive passaging and then examined these stemness markers (OCT4, SOX2, and c-MYC) and the multilineage differentiation capability of MSCs when treated with FN, FGF2, and BMP4. Furthermore, we focused on the identification of molecular signatures for stemness maintenance of MSCs via metabolic and proteomic cues in this process. The findings from this study will be of significance in facilitating the application of MSCs and extending the field of clinical applications for cell-based therapy in regenerative medicine.

## Materials and methods

### Cell culture and treatments

Bone marrow-derived MSCs (BMSCs) were isolated and cultured as described previously [1]. Briefly, the bone marrow was obtained from patients (50–60 years old) undergoing elective knee replacement surgery at the Prince Charles Hospital after informed consent was given (*n* = 5). The procedure was approved by the Office of Research Ethics and Integrity, Queensland University of Technology (ethics approval number 1400001024). Lymphoprep (Axis-Shield PoC AS, Oslo, Norway) was added to isolate the mononuclear cells from the bone marrow by density gradient centrifugation. The obtained cells were maintained in Dulbecco’s modified Eagle’s medium (DMEM; Gibco®, Life Technologies Pty Ltd., Australia) supplemented with 10% fetal bovine serum (FBS; In Vitro Technologies, Australia) and 1% (v/v) penicillin/streptomycin (P/S; Gibco®, Life Technologies Pty Ltd., Australia) at 37 °C in a humidified CO_2_ incubator containing 5% CO_2_. The culture medium was changed every 3 days, and unattached hematopoietic cells were removed by the media changes. The confluent cells were expanded and only early passages (passage 1–5) of cells were used in this study.

To determine the effect of FN (R&D Systems, Minneapolis, MN, USA), FGF2 (R&D Systems, Minneapolis, MN, USA) and/or BMP4 (R&D Systems, Minneapolis, MN, USA) on the expression of OCT4, SOX2, and c-MYC, six different experimental groups were designed in this study as follows: (1) a control group where BMSCs were cultured with DMEM supplemented with 10% FBS, (2) BMSCs cultured on 5 μg/mL FN-coated plates with DMEM supplemented with 10% FBS, (3) BMSCs cultured with DMEM supplemented with 2 ng/mL FGF2 and 10% FBS, (4) BMSCs cultured on FN-coated plates and DMEM supplemented with 2 ng/mL FGF2 and 10% FBS, (5) BMSCs cultured on FN-coated plates and DMEM supplemented with 10 ng/mL BMP4 and 10% FBS, and (6) BMSCs cultured on FN-coated plates and DMEM supplemented with 2 ng/mL FGF2, 10 ng/mL BMP4, and 10% FBS.

### Quantitative real-time reverse-transcription polymerase chain reaction (qRT-PCR)

Total RNA of cultured cells at passage 5 from six different experimental groups was isolated with TRIzol® reagent (Life Technologies Pty Ltd., Australia) as per manufacturer’s instructions. Reverse transcription was performed using DyNAmo™ cDNA Synthesis Kit (Genesearch Pty Ltd., Australia). SYBR Green qPCR Master Mix (Life Technologies Pty Ltd., Australia) was used for detection, and *OCT4, SOX2*, and *c-MYC* mRNA expression were assayed on the ABI PRISM® 7500 FAST Sequence Detection System (Applied Biosystems, California, USA). The relative gene expression was obtained by normalizing the mean cycle threshold (Ct) value of each target gene with the Ct value of the house-keeping gene glyceraldehyde-3-phosphate dehydrogenase (*GAPDH*). The corresponding primer sequences of the reference gene and the target genes were designed based on cDNA sequences from the NCBI Sequence Database (Table 1).

**Table 1:** Primer sequences used in a quantitative real-time polymerase chain reaction

GENE	Primer
*OCT4*	Forward: 5′-GCTCGAGAAGGATGTGGTC-3′
	Reverse: 5′-ATCCTCTCGTTGTGCATAGTCG-3′
*SOX2*	Forward: 5*′*- CACTGCCCCTCTCACACATG-3*′*
	Reverse: 5*′*- CCCATTTCCCTCGTTTTTCTT-3*′*
*c-MYC*	Forward: 5*′*- GGCTCCTGGCAAAAGGTCA-3′
	Reverse: 5*′*- AGTTGTGCTGATGTGTGGAGA-3*′*
*ACAN*	Forward: 5*′*- AGACTTGGTGGGGTCAG-3*′*
	Reverse: 5*′*- GATGTTTCCCACTAGTG-3*′*
*OPN*	Forward: 5*′*- CTGAGGCTGAGAATACCACACTT-3*′*
	Reverse: 5*′*- GGTGATGTCCTCGTCTGTA-3*′*
*PPAγ2*	Forward: 5*′*-CTGTTGACTTCTCCAGCA-3*′*
	Reverse: 5*′*-GTCAGCGGACTCTGGA-3*′*
*GAPDH*	Forward: 5*′*-TCAGCAATGCCTCCTGCAC-3*′*
	Reverse: 5*′*-TCTGGGTGGCAGTGATGGC-3*′*

### Western blot analysis

The BMSCs at passage 5 were cultured on FN-coated plates and DMEM supplemented with 2 ng/mL FGF2, 10 ng/mL BMP4, and 10% FBS. The whole-cell lysates of stimulated BMSCs were collected after 3 days of culture. Equivalent amounts of diluted protein samples (15 μg) were resolved by 10% SDS-polyacrylamide gel electrophoresis and transferred onto nitrocellulose membranes. Bands were detected with polyclonal antibodies (1:1000) against OCT4 (Abcam, USA), SOX2 (Abcam, USA), and c-MYC (Abcam, USA). a-Tubulin (1:5000; Abcam, USA) was used as a loading control. The protein bands were visualized using the Odyssey Infrared Imaging System (LI-COR Biotechnology, Nebraska, USA). The relative intensity of protein bands compared with α-Tubulin was quantified using Image Studio™ Software.

### Immunofluorescence staining

BMSCs in the FN + FGF2 + BMP4 treatment group and untreated control group at passages 3 and 5 were cultured in Nunc™ chamber slides (Thermo Fisher Scientific, USA). Briefly, the cells were fixed with 4% (w/v) paraformaldehyde (PFA) for 15 min and then permeabilized with 0.1% Triton X-100 in PBS for 10 min, incubated in 3% bovine serum albumin in PBS for 30 min and with the primary monoclonal anti-OCT4 antibody (1:100), anti-SOX2 antibody (1:100), and anti-c-MYC antibody (1:100) at 4 °C overnight. Finally, all samples were rinsed and then incubated with fluorochrome labeled (Alexa Fluor 488) secondary antibody (1:150, Invitrogen, USA). Images were visualized and captured using a fluorescence microscope (Carl Zeiss Microimaging Gmbh, Gottingen, Germany).

### In vitro differentiation of BMSC populations

Differentiation capabilities of the FN + FGF2 + BMP4 treatment group and untreated control group at passage 5 were determined by stimulating cells in selective differentiation media for osteogenic, chondrogenic, and adipogenic lineage.

#### Chondrogenic differentiation

Cells at passage 5 from both groups were differentiated into chondrocytes according to a previously described micro-mass pellet culture [35]. The cell pellets were cultured in a chondrogenic medium. After 21 days, the cell pellets were stained with Alcian blue for evidence of proteoglycan deposition, and the mRNA expression of *aggrecan (ACAN)* was measured.

#### Osteogenic differentiation

Osteogenic differentiation was induced on a monolayer of confluent populations with complete media supplemented with 50 mM ascorbic acid, 10 mM β-glycerol phosphate, and 100 nM dexamethasone (Sigma-Aldrich, Castle Hill, NSW, Australia). After 21 days, the cells were stained with Alizarin Red S to observe calcium depositions and the mRNA expression of *osteopontin (OPN)* was measured.

#### Adipogenic differentiation

Cells at passage 5 for both groups were differentiated into adipocytes as previously described [36]. Lipid droplets were stained with Oil Red O and the mRNA expression of *peroxisome proliferator-activated receptor γ2 (PPAγ2)* was measured.

### Metabolomic and proteomic characterization

Details of the procedure were provided in the Supplementary Materials and Methods.

### Genomic biological prediction of target genes regulated by OCT4, SOX2, and c-MYC

The *Homo sapiens* genes regulated by three transcription factors, POU5F1/OCT4, SOX2, and MYC/c-MYC, were predicted by the Gene Transcription Regulation Database (GTRD, http://gtrd.biouml.org/). Ensembl Database (https://rest.ensembl.org) was used for the annotation process of the data from GTRD. Venn diagrams in the R package were utilized to compare 3 datasets from the transcription factors, among which the co-regulated coding-protein genes were selected. Based on these genes, functional enrichment analysis in terms of pathway analysis was performed by the cluster profiler R package, and Venn analysis was performed with the known metabolic pathways identified from the metabolomic characterization. In addition, the co-regulated genes and 21 differential genes based on proteomic analysis were further analyzed using Venn analysis. Furthermore, we explored the potential regulatory feedback between the metabolic and proteomic data through analyzing their common KEGG pathways and subsequently correlated metabolites and differential proteins in the common pathways.

### Statistical analysis

All experiments were performed in triplicate. The results obtained from this study were expressed as mean ± SD (standard deviations). Statistical analysis was performed using GraphPad Prism 7 (Version 7.02) for Windows (GraphPad Software Inc., USA). Statistical differences between groups were determined by one-way ANOVA with Bonferroni’s multiple comparison tests. *p* < 0.05 was considered statistically significant.

## Results

### Expression pattern of stemness marker in BMSCs with successive passaging

**Fig. 1:**
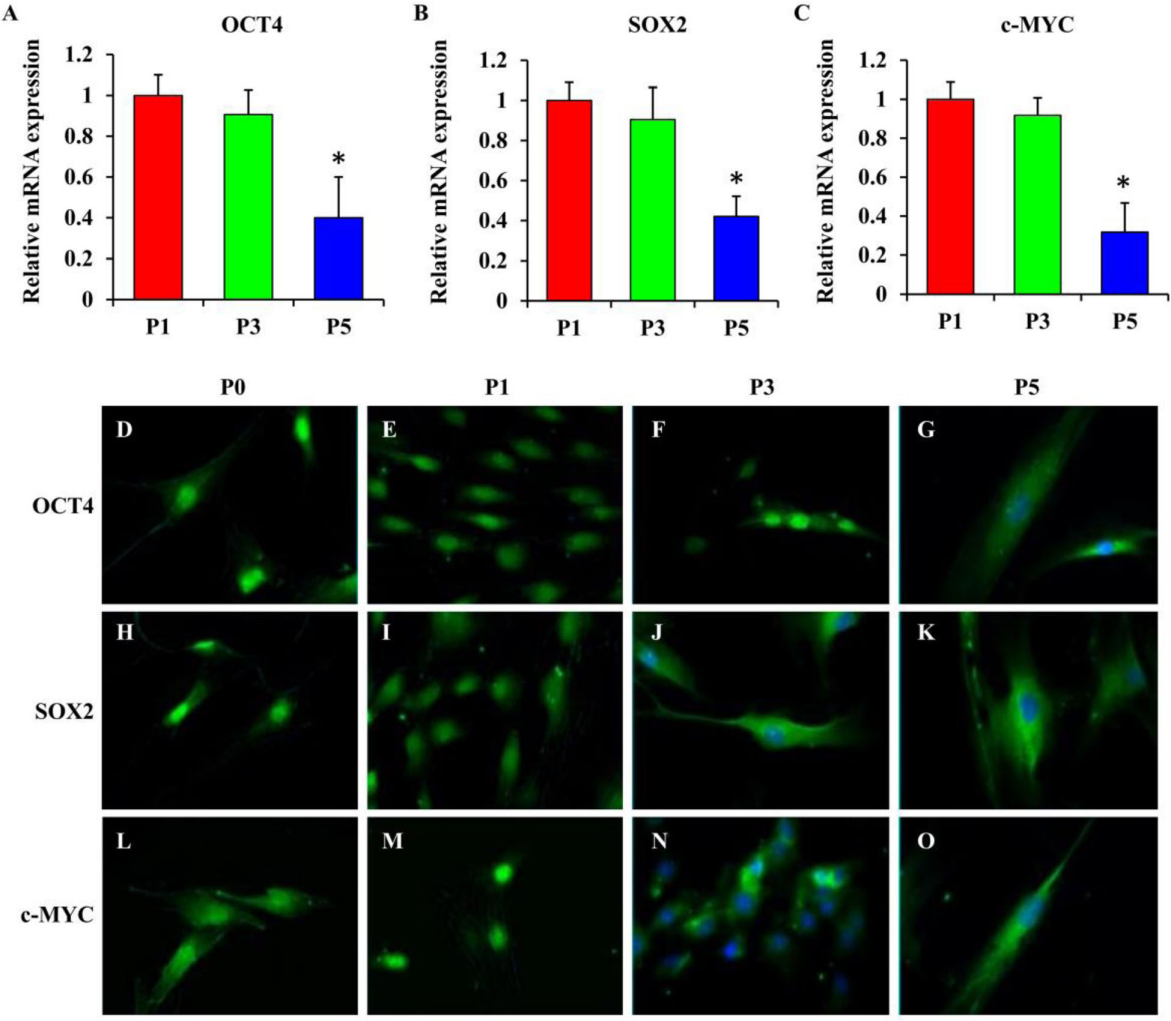
Expression of OCT4, SOX2, and c-MYC in BMSCs at various passages. **a**–**c** The mRNA expressions of *OCT4*, *SOX2*, and *c-MYC* were downregulated in BMSC cultures at passage 5 compared to those at passages 1 and 3. The primary antibody of each target protein OCT4 (**d**–**g**), SOX2 (**h**–**k**), and c-MYC (**l**–**o**) was detected with Alexa Fluor 488 (green stain) at various passages. Statistical significance was accepted at *p < 0.05

The mRNA expressions of *OCT4, SOX2*, and *c-MYC* were downregulated in culture from passage 1 to passage 5 (Fig. 1a–c). Among all groups, the cells at passage 5 had the most significant reduction in stemness marker expressions compared to other groups (*p* < 0.05). Immunofluorescence staining revealed that OCT4 (Fig. 1d–g), SOX2 (Fig. 1h–k), and c-MYC (Fig. 1l–o) while detected in the nucleus as early as the primary culture exhibited cytoplasmic expression with subsequent passaging.

### Manipulation of microenvironment counteracted the change of stemness marker expression patterns

**Fig. 2:**
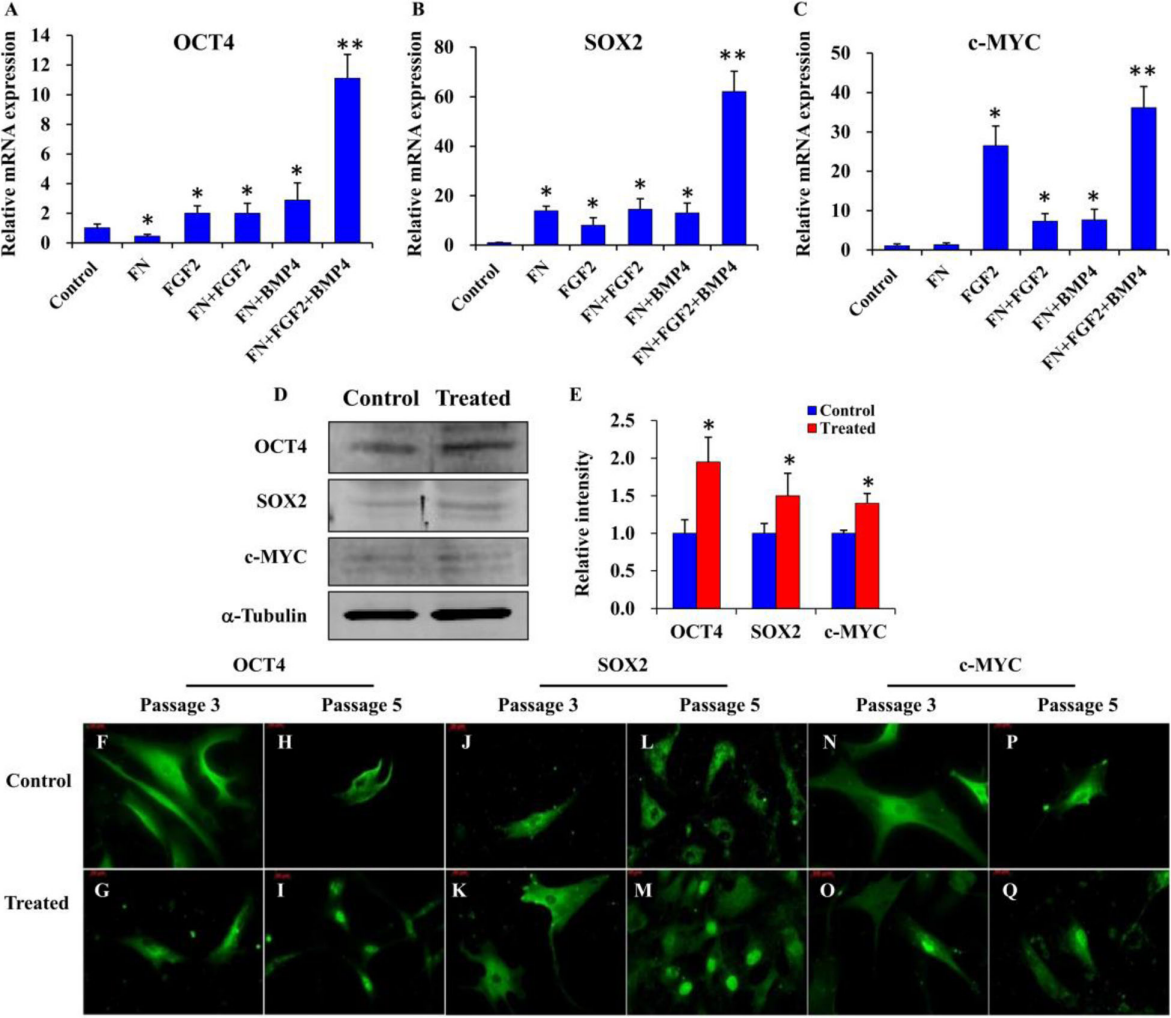
The expression and translocation pattern of OCT4, SOX2, and c-MYC with the stimulation of FN + FGF2 + BMP4. **a**–**c** All treatments transiently induced the expressions of *OCT4*, *SOX2*, and *c-MYC*, and the induction effect of FN + FGF2 + BMP4 was most obvious. **d**, **e** Treated cells with FN + FGF2 + BMP4 enhanced the protein expression of OCT4, SOX2, and c-MYC. The primary antibody of each target protein OCT4 (**f**–**i**), SOX2 (**j**–**m**), and c-MYC c (**n**–**q**) was detected with Alexa Fluor 488 (green stain). Nuclear translocation of the target transcription factors was observed in all treated samples (FN + FGF2 + BMP4) at passage 5. Statistical significance was accepted at *p < 0.05 or **p < 0.001

The qRT-PCR analysis of BMSCs at passage 5 showed that the mRNA expressions of *OCT4* (Fig. 2a), *SOX2* (Fig. 2b), and *c-MYC* (Fig. 2c) were all significantly upregulated relative to the control in the experimental groups, FGF2, FN+ FGF2, and FN + BMP4-treated groups, respectively (*p* < 0.05). Importantly, the most significant upregulation occurred in BMSC cultures stimulated with FN + FGF2 + BMP4 (*p* < 0.001) (Fig. 2a–c). Western blot analysis showed similar expression patterns to the qRT-PCR results. Expression of all the stemness markers (OCT4, SOX2, and c-MYC) was enhanced in the FN + FGF2 + BMP4 group (Fig. 2d, e). Immunofluorescence staining revealed that OCT4 (Fig. 2f–i), SOX2 (Fig. 2j–m), and c-MYC (Fig. 2n–q) while detected in the cytoplasm at passages 3 and 5 in the control group exhibited nuclear expression at passage 5 in the FN + FGF2 + BMP4-treated group, indicating cytoplasmic to nuclear translocation.

### Manipulation of microenvironment increased differentiation potential of BMSCs

**Fig. 3:**
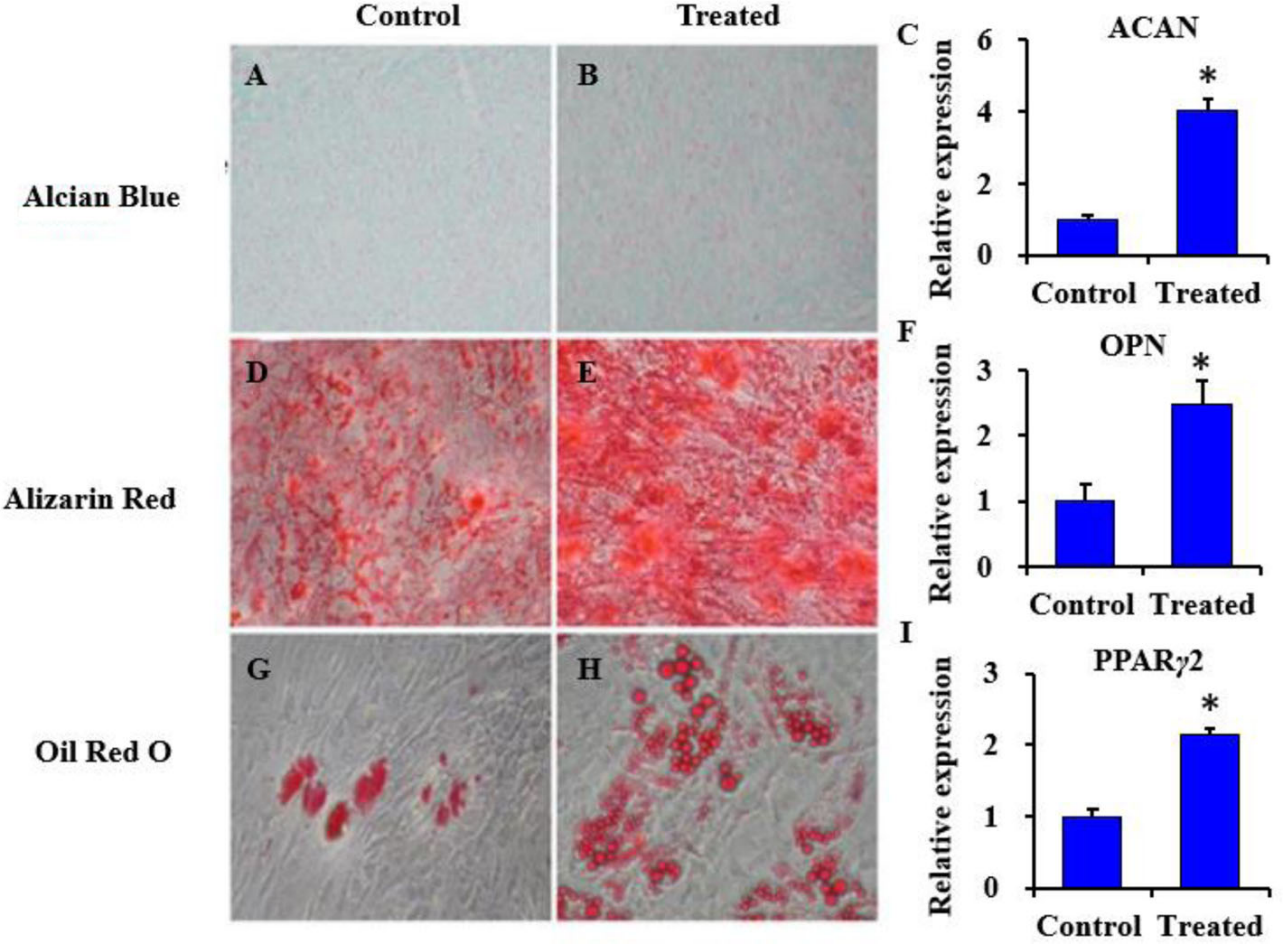
The differentiation potential of control and treated BMSCs. Treated (FN + FGF2 + BMP4) and control BMSCs at passage 5 were induced in chondrogenic, osteogenic, and adipogenic medium for 21 days, followed by staining and qRT-PCR analysis (**a**–**h**). Strong staining for all three lineages was observed in treated BMSCs. The qRT-PCR analysis for **c** *ACAN*, **f** *OPN*, and **i** *PPARγ2* revealed higher expression of lineage-specific markers in treated BMSCs. Statistical significance was accepted at *p < 0.05

After 21 days of multi-lineage differentiation induction, the treated BMSCs at passage 5 appeared to be more multi-potent as revealed by the staining pattern and differentiation transcript expression. Alcian blue staining was more prominent in the treated samples compared with the untreated control group (Fig. 3a, b). The *ACAN* gene transcript expression was significantly higher in the treated samples (*p* < 0.05) (Fig. 3c). When cultured in osteogenic media, both groups exhibited mineralization and calcium deposition (Fig. 3d, e). The treated samples were almost completely covered with calcium deposits as revealed by Alizarin Red S staining (Fig. 3e). *OPN* expression corresponding to osteogenic differentiation was higher in the treated group (*p* < 0.05) (Fig. 3f). For adipogenic differentiation, treated BMSCs after 21 days of induction exhibited a larger number of lipid droplet clusters compared to the untreated control group (Fig. 3g, h). Similarly, *PPARγ2* expression was higher in the treated group (*p* < 0.05) (Fig. 3i). Thus, FN + FGF2 + BMP4 promoted the multi-lineage differentiation of BMSCs.

### Manipulation of microenvironment facilitated metabolic alterations

**Fig. 4:**
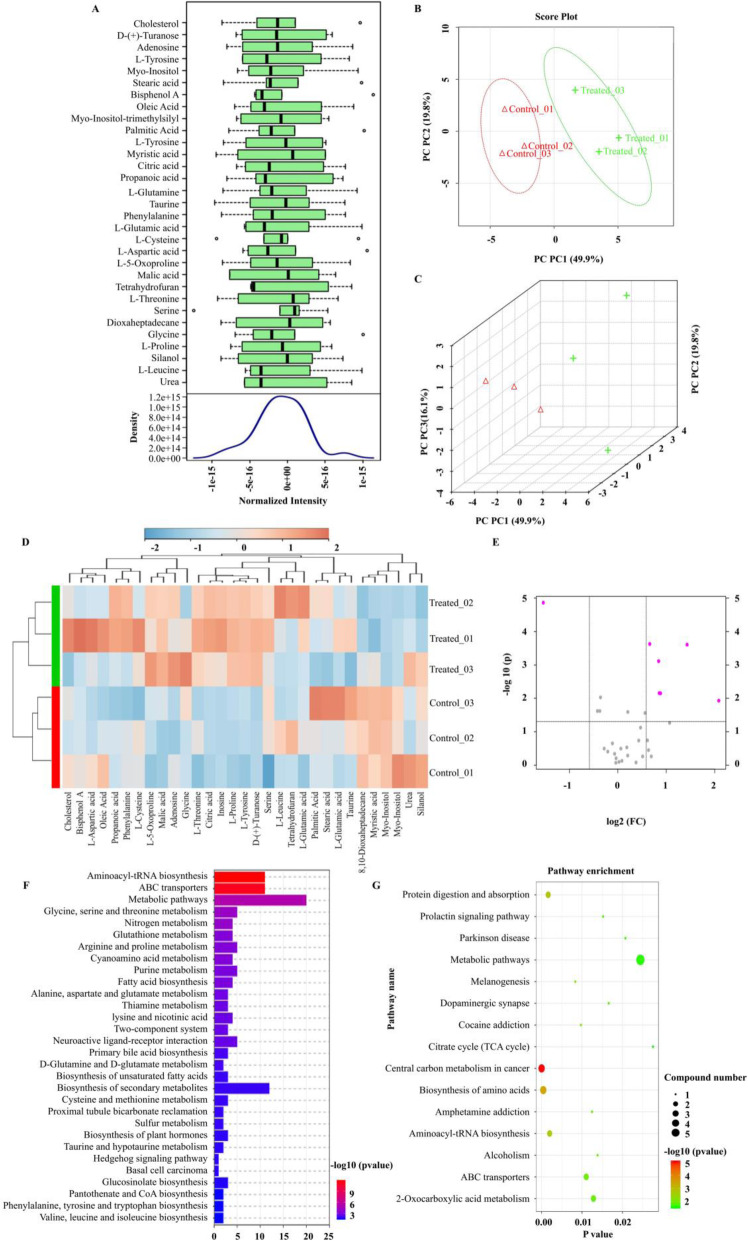
GC-MS analysis of treated BMSCs compared to untreated BMSCs. **a** Box plots and kernel density plots after normalization. **b**, **c** 2 and 3D score plot between all samples. The explained variances were shown in brackets. **d** Heatmap showed 31 metabolites identified by GC-MS. **e** Volcano plots demonstrated differential abundance between two groups. The vertical lines corresponded to a 1.5-fold increase and decrease in abundance and the horizontal line represents a *p* value of 0.05. The colored dots in the plot represent the metabolites that exhibited statistically significant differential abundance compared to controls. **f** KEGG pathway annotation of the analytes where the horizontal axis represents the number of metabolite pathway associated genes. **g** KEGG pathway annotation of the differentially abundant analytes. The horizontal axis indicated *p* value and vertical ordinates were the terms of the pathways. The size of the node indicates the number of metabolite pathway-associated genes matched in the pathways. The degree of color represents −log_10_ (*p* value): Logarithmic conversion of Fisher’s exact test *p* value, indicating the significance of pathway correlations

GC-MS was utilized to determine the metabolic cues involved in the maintenance of BMSCs. Row-wise normalization allowed general-purpose adjustment for differences among samples thereby allowing users to manually adjust concentrations based on biological inputs. The density plot analysis based on all samples, indicated that the metabolite data distribution conformed to normal distributions (Fig. 4a). The 2D and 3D scores plot indicated distinct clustering between samples of the control or treatment groups, which met the experimental requirements and could be used for subsequent analysis (Fig. 4b, c). The 31 identified analytes are presented in Table S1. The clustering results were shown in the form of a heatmap (Fig. 4d). Using volcano plot analysis, we also identified 7 differentially abundant metabolites that had a greater than 1.5-fold change in the treated BMSCs compared with the control (Myo-Inositol, L-Proline, D-(+)-Turanose, L-Tyrosine, Malic acid, Citric acid, Inosine) (Fig. 4e). The functional annotations of the abundant metabolites were further examined using KEGG Ontology assignments to classify their relative influence on various biochemical pathways (Fig. 4f, g). Primarily, this included increases in pathways that play a role in metabolic activity, energy production, and potentially protein production.

### Proteomic alterations for stemness maintenance of BMSCs

**Fig. 5:**
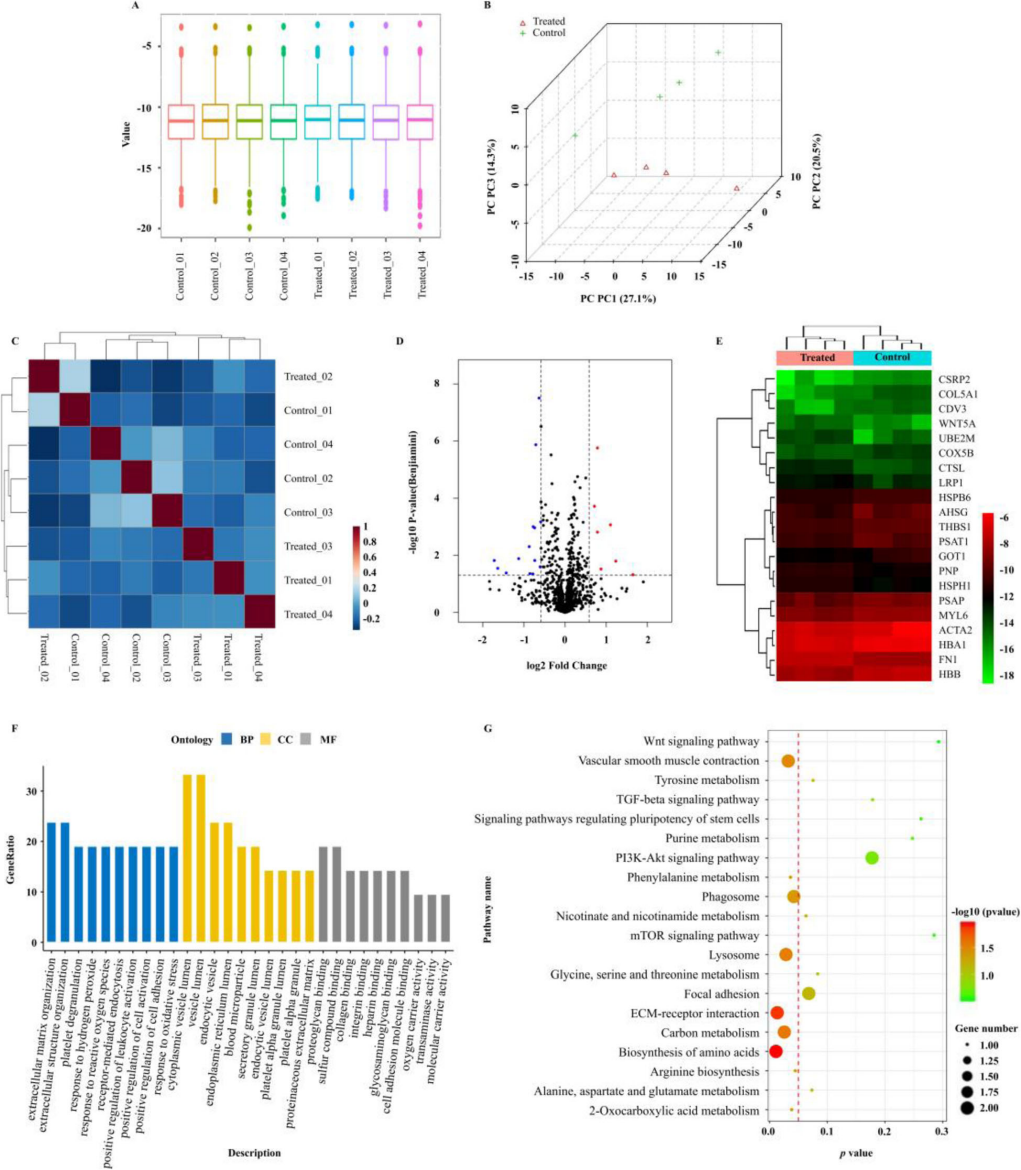
Proteomic characterization of treated BMSCs compared to untreated BMSCs. **a** Box plots to visualize the distributions of a dataset. The box plots in the same figure indicated that the distribution of the intensities among all samples was nearly the same. **b** 3D score plot for all samples. The explained variances were shown in brackets. **c** Hierarchical clustering was performed with the h.clust function in a statistical package, and the clustering result was shown in the form of a heatmap. **d** Correlation analysis could be used to visualize the overall correlations between different samples. **e** Volcano plots were used to demonstrate differential abundance between two groups. The vertical lines corresponded to 1.5-fold up and down and the horizontal line represented a *p* value of 0.05. The colored dots in the plot represented the differentially abundant proteins with statistical significance. **f** Heatmap showed 21 differentially abundant proteins among samples (high relative abundance in red, and low relative abundance in green or black). **g** Gene ontology (GO) analysis of differentially abundant proteins. The vertical axis represented the gene ratio (the ratio of the gene count in GO terms to the total differentially expressed genes count) and the horizontal axis described the enrichment components. BP biological process, CC cellular component, MF molecular function. **h** KEGG significant enrichment analysis for the differentially abundant proteins. The horizontal axis indicated *p* value and vertical ordinates were the terms of the pathways. The size of the node indicated the number of genes matched in the pathways. The degree of color represented −log_10_ (*p* value): Logarithmic conversion of Fisher’s exact test *p* value, indicating the significance of pathway correlations

LC-MS/MS was performed to explore the altered proteomes involved in the maintenance of BMSCs. The box plot in Fig. 5a indicated that the distribution of the intensities among all samples was nearly the same. The 3D scores plot indicated a close distance between samples in the same groups, which met the experimental requirements and can be used for subsequent analysis (Fig. 5b). Figure 5c showed the correlation heatmap. The Volcano Plots in Fig. 5d showed differential abundance between samples. We identified 21 differentially abundant proteins that had a greater than 1.5-fold change in the treated BMSCs compared with the control (Table S2 & Fig. 5e). Among these, 7 proteins were upregulated, whereas 14 proteins were downregulated (Table S2). We further determined the functional annotations of the differentially abundant proteins through gene ontology analysis (Fig. 5f). These data indicated that the differentially abundant proteins were associated with extracellular matrix/structure organization, the production of cytoplasmic vesicles, and the proteoglycan or sulfur compound binding. Additionally, KEGG significant enrichment analysis showed differentially abundant proteins involved in multiple signal transduction, including metabolic pathways, autophagy-related pathways, and signaling pathways regulating pluripotency of stem cell (Fig. 5g).

### Biological analysis revealed a potential relationship between metabolic and proteomic cues involved in stemness maintenance

Target genes regulated by 3 transcription factors were identified (Table S3) and 31,850 co-regulated genes (including protein genes and non-coding RNAs) were found by Venn analysis (Table S3 & Fig. S1). Out of the 31,850 co-regulated genes, 31,526 were present in the Ensembl Database, among which 16,129 were coding-protein genes (16,126, excluding repeated ones) (Table S3). These genes were associated with a variety of signaling pathways, among which 17 were known metabolic pathways based on the metabolic results via Venn analysis (Table S4). Additionally, Venn analysis was further performed to correlate these 16,126 co-regulated genes and the 21 genes with noted differences from the proteomic analysis. The results showed that a total of 20 genes were co-expressed. The gene outside of the intersection was HBB, which is a potential regulatory gene of SOX2 and c-MYC (Fig. S2). All these data implied a reciprocal communication between metabolic/proteomic cues of MSCs and their stemness. Furthermore, we identified five common KEGG pathways between the metabolic and proteomic analysis (Table S5), among which the metabolites and differential proteins were correlated in the same pathways (Fig. S3 - Fig. S7).

## Discussion

BMSCs are a resource for autologous and allogeneic cell therapies in regenerative medicine [1, 3, 37]. However, BMSCs senesce with prolonged culture in vitro and undergo a decline in stemness and progenitor cell functions such as proliferation and differentiation, thereby limiting their therapeutic applications [6–8]. Some researchers have reported various in vitro treatments that may improve the performance of aged BMSCs [7, 8, 26–28]. Thus, improving the conditions of cell culture and discovering potential signaling cues that regulate BMSC aging may help to maintain the competency of BMSCs.

As noted in recent research, cell reprogramming leading to pluripotency is a gradual process involving the sequential reactivation of pluripotency markers such as OCT4, SOX2, and c-MYC [12, 31, 38–41]. In the present study, following the isolation and characterization of BMSCs, we assessed the stemness markers (OCT4, SOX2, and c-MYC) of these cells and found that they decreased with successive passaging. Notably, the molecules FN, FGF2, and BMP4 could rescue the stemness loss with an individual or synergistic effect, among which the simultaneous introduction of FN, FGF-2, and BMP4 significantly increased the expression of OCT4, SOX2, and c-MYC. Thus, we anticipate that the manipulation of the microenvironment with the ECM component and growth factors will be useful concerning the reverse of the aging BMSCs.

Based on our immunofluorescence staining results, OCT4 along with SOX2 and c-MYC were mainly localized in the nucleus and gradually lost their nuclear localization while being expressed in the cytoplasm of BMSCs after passaging in vitro, indicating the loss of their functional role during this process. Of note, when treated with FN, FGF2, and BMP4, the location of stemness markers translocated from the cytoplasm to nuclear. Interaction of these key transcription factors leads to the establishment of a regulatory loop which controls the self-renewal and differentiation potential of stem cells, implying that the “cocktail treatment” of combined stimulating factors may keep cells in a state of active reprogramming and is critical for maintaining stemness. Consistently, those treated BMSCs formed significant Alcian blue-positive pellets, Alizarin Red-positive mineralized nodules, and Oil Red O-positive lipid droplets. Therefore, the treated BMSCs hold much promise in regenerative medicine, and identification of the underlying molecular mechanisms is an avenue for further research.

Recent advancement of various high-throughput experimental techniques makes it feasible to integrate large amounts of data into a coherent quantitative picture of cell fate control. Once thought to be a mere consequence of the state of a cell, metabolism is now known to play a pivotal role in dictating whether a cell proliferates, differentiates or remains quiescent [42–46]. GC-MS is a predominant tool in the detection of metabolites due to its ability to explore the unique fingerprints that specific cellular processes leave behind [47, 48]. It can analyze metabolite abundance to reveal active metabolic pathways which allow for unique biomarker identification [47, 49–51].

Recent studies have demonstrated that nuclear reprogramming of stem cells through the induction of transcription factors associated with dramatic metabolic remodeling [42–46]. Among 7 differentially abundant metabolites, Proline, Citric acid, Tyrosine and Malic acid are intermediate metabolites of the energy-generating pathways, such as the tricarboxylic acid cycle (TCA cycle), the glutamine metabolism and the proline regulatory axis. All these pathways play a role in metabolic remodeling, which can fuel maintenance of pluripotent state [43, 44]. Besides, Myo-Inositol [52], and Inosine [53] can regulate the antioxidant defenses. As the cellular redox status plays an important role in stem cell biology [54], these two altered metabolites may help preserve stem cell functionality. To understand the biological functions of the abundant metabolites further, KEGG Ontology assignments were conducted to classify their relative influence on various biochemical pathways. The functional identity of the target genes confirmed that these metabolites may have important implications in the maintenance of BMSCs through their effects on metabolic activity, energy production, and potentially protein production. Interestingly, based on the bioinformatics analysis, we identified 17 common pathways in the relation network among these metabolites-associated pathways and the biological pathways associated with 3 transcription factors. All these results suggested that the introduction of FN, FGF-2, and BMP4 can regulate the metabolic pathways, thereby driving cell fate conversions by regulating the transcriptional identity of the stem cells.

Concurrently, the LC-MS proteomic technique was also utilized in our study to characterize the protein complements involved in stemness maintenance. This type of study generates a snapshot of the environment’s proteins and provides an overall view of the potential biochemical pathways being utilized by the cells [55, 56]. Our study identified 21 differentially abundant proteins, among which 20 were predicted to be the targets of 3 transcription factors (OCT4, SOX2, and c-MYC), indicating that the proteins from proteomic analysis participated in the pluripotent fate control of BMSCs. To further understand the orchestrating roles of these 21 abundant proteins in the pluripotency of BMSCs, their potential target gene functions were illustrated using gene ontology and KEGG pathway analysis. The ontology analysis data indicated that 21 differentially abundant proteins were associated with extracellular matrix/structure organization, the production of cytoplasmic vesicles, and the proteoglycan or sulfur compound binding. Additionally, KEGG pathway analysis revealed that target genes of the 21 differentially expressed proteins were involved in multiple signal transductions, including metabolic pathways, autophagy-related pathways, and signaling pathways regulating pluripotency of stem cells. Of note, some of these were consistent with our metabolic results which were mentioned above; thus, we performed bioinformatics analysis to further explore the correlation between metabolic and proteomic profiling. According to our results, we identified five common KEGG pathways between these two analysis techniques. These findings suggest that the importance of metabolism and proteome networks and their reciprocal communication in the preservation of stemness and the modulation of cell-fate decision making.

Proteomic analysis of MSCs could provide detailed information into what processes are occurring in the cells and can be used to monitor their growth and stemness. Concordantly, metabolomic studies can provide a more acute perspective on the dynamic changes of an environment, as the flux of metabolites can provide information on the activity of the cells [42, 47]. Determination of both the metabolomic and proteomic markers could help us fully understand the biological status of the MSCs and provide significant insights into how to maintain their stem phenotype. Further research needs to be conducted to confirm how these metabolic intermediates or proteins contribute to the stemness maintenance during nuclear reprogramming. While GC-MS and LC-MS are highly reliable analytical techniques, using them in tandem with other high-throughput analytical techniques would obtain a more accurate analysis of the results. Despite the limitations of this research due to using only one method of metabolic or proteomic analysis, this study demonstrates that the manipulation of the microenvironment can lead the cells with high passage numbers to a more primitive cell state, which may be related to a specific metabolomic and proteomic profile.

## Conclusion

The present study has demonstrated that endogenous expression of stemness markers (OCT4, SOX2, and c-MYC) can be induced by modulation of culture conditions (FN, FGF-2, and BMP4) and deepen our understanding of the contribution made by metabolic and proteomic cues to the regulation of BMSC stemness. Further investigations into the metabolite-protein dynamics and the underlying molecular mechanisms hold great therapeutic promise to direct stem cell fate for tissue regeneration and the development of novel strategies to combat degenerative disorders.

## Supplementary Information

**Additional file 1: Supplementary Materials and Methods**.

**Additional file 2: Table S1.**

**Additional file 3: Table S2.**

**Additional file 4: Table S3.**

**Additional file 5: Table S4** and **Table S5.**

**Additional file 6: Supplementary Figures.**

## Data Availability

The datasets used and/or analyzed during the current study are available from the corresponding author on reasonable request.

## Abbreviations

MSCs: Mesenchymal stem cells
FN: Fibronectin
FGF2: Fibroblast growth factor 2
BMP4: Bone morphogenetic protein 4
BMSCs: Bone marrow derived MSCs
GC-MS: Gas chromatography–mass spectrometry
LC-MS/MS: Liquid chromatography–tandem mass spectrometry
TCA cycle: Tricarboxylic acid cycle
DMEM: Dulbecco’s modified Eagle’s medium
FBS: Fetal bovine serum
P/S: Penicillin/streptomycin
qRT-PCR: Quantitative real-time reverse-transcription polymerase chain reaction
GAPDH: Glyceraldehyde-3-phosphate dehydrogenase
PFA: Paraformaldehyde
ACAN: Aggrecan
OPN: Osteopontin
PPAγ2: Peroxisome proliferator activated receptor γ2
GTRD: Gene Transcription Regulation Database
GO: Gene ontology
