# Supplementary material for: Effect of fibronectin, FGF-2, and BMP4 in the stemness maintenance of BMSCs and the metabolic and proteomic cues involved
Source: Stem Cell Res Ther. 2021 Mar 6;12:165. doi: 10.1186/s13287-021-02227-7 (PMC7936451; doi:10.1186/s13287-021-02227-7)
Supplement: Supplementary file 1 — Additional file 1: Supplementary Materials and Methods. [file 13287_2021_2227_MOESM1_ESM.docx]

**Supplementary Materials and Methods**

**1. Metabolomic characterization**

### *1.1 Sample harvest*

The cells in the treatment group were cultured on FN-coated plates and with the addition of FGF2 and BMP4 and the control cells were cultured in normal medium. After stimulation for three days, the cells were washed with PBS and 100 µL of methanol was added to harvest the cells. Cell samples were transferred to Eppendorf tubes and beads were used to homogenize the samples.

### *1.2 Metabolite derivatization*

The positive control was prepared by separating stocks of 1 mg/mL of glycine, ribitol, glucose and sucrose. Each stock was diluted one hundred times with LCMS grade water to a final concentration of 10 ppm. Six microliters of this solution was then aliquoted into prepared Eppendorf tubes with glass inserts. Twenty-four microliters of methanol was used as a negative control group. Twenty-four microliters of the harvested biological sample was aliquoted respectively. The samples were then vacuum-concentrated to dryness without heating (37 °C, 1500 RPM for 30 min).

### *1.3 Oximation and silyation*

Twenty microliters of Meox was added to each glass insert. Forty microliters of BSTFA/1% TMCS was added and incubated at 37 °C, 500 RPM for 40 min. Samples were then transferred into autosampler vials and analyzed by gas chromatography mass spectrometry (GC-MS).

### *1.4 Data analysis*

Derivatized metabolite samples were placed into the Shimadzu Triple Quadrupole 8040 GC-MS platform. Metabolites were identified with Xcailbur using the NIST Standard Reference Database. Once metabolites had been identified and areas calculated the Golm Metabolome Database and the NIST Database were used to identify analytes and metabolites to understand the current state of the MSCs. Hierarchical clustering was performed to distinguish between control and the treated group. Metabolite enrichment analysis was performed based on the Kyoto Encyclopedia of Genes and Genomes (KEGG) and MBRole (<http://csbg.cnb.csic.es/mbrole2/>). After the enrichment analysis, the negative logarithm of the *p*-values was calculated based on 10 to clearly display the significance of each pathway in a histogram.

**2. Proteomic characterization**

### *2.1 Protein Collection*

The bMSCs in the treatment group were cultured on FN-coated plates and with the addition of FGF2 and BMP4 and the control cells were cultured in normal medium. After stimulation for three days, cells were washed with PBS, prior to addition of lysis buffer, containing 4% sodium dodecyl sulphate (SDS), 0.1 % Tris/HCl (pH 7.6), 0.1 M dithiothreitol (DTT) and protease inhibitor cocktail, to the culture flasks. The cell lysates were collected and vortexed for 2 minutes, heated to 80 ºC for 5 minutes and sonicated for 10 minutes to disrupt cellular membranes and liberate protein. The free protein was concentrated using Amicon Ultra-15 3KDa Centrifugal filter devices by centrifugation at 4000 ×g in a swing bucket centrifuge (Allegra® X-15R Centrifuge) for 20 minutes. The protein content in the retentate of each sample was quantified using the Pierce Coomassie (Bradford) Protein Assay kit as per the manufacturer’s instructions.

### *2.2 Protein Processing for Library Generation*

### *2.2.1 Pooled Sample*

A pooled sample was created by combining an aliquot of each sample together in a single Protein Lo-Bind Eppendorf tube (Eppendorf, Hamburg, Germany) to a final 0.7mg of total protein. The protein concentration in the pooled sample was quantified using a Bradford Assay as per the manufacturer’s instructions.

### *2.2.2 Iso-electric Focusing*

Isoelectric focusing was used to separate the proteins based on their isoelectric properties. The instrument (Agilent 3100 OFFGEL Fractionator) was set up by first removing the protective backing from the IPG strip, consisting of a linear pH gradient of 3 – 10, and placing the strip into the tray gel side facing up. The welled frame was clipped into place on the gel before each of the wells received 20 μl of IPG Strip Rehydration Solution (0.48 mL OFFGEL Stock Solution (1.25X; 8.4 M Urea, 2.4 M Thiourea, 0.78 M Dithiothreitol, 12 % glycerol + ampholytes) in 0.12 mL MilliQ H2O) ensuring the gel strip was covered. A pair of electrode pads were wetted with the rehydration buffer and placed on either end of the IPG strip. An aliquot of the pooled sample (400μg of protein) was diluted and mixed with the OFFGEL stock solution. After 15 minutes, the sample solution was transferred evenly among the wells and the cover seal was placed over the frame. The electrode pads were rewetted with rehydration buffer prior to the tray being assembled on the instrument platform. Mineral oil was applied to the anode and cathode pads. After 1 minute, additional mineral oil was reapplied to both ends of the strip. Both electrodes were attached to the tray and current was applied to the samples for 71 kV.hours. The focused proteins were maintained under 20 μA current until they were subjected to FASP as described below.

### *2.2.3 LDS-PAGE*

Lithium dodecyl sulphate polyacrylamide gel electrophoresis (LDS-PAGE) was used for separation of pooled sample proteins. Briefly, an aliquot of the pooled sample containing 30 μg of protein was mixed with 4X NuPAGE® LDS sample buffer, 100 mM DTT and HPLC water to a final volume of 10 μL then heated at 90 ºC for 5 minutes. The sample was loaded into a 10-well precast 4-12 % Bis-Tris gel and subjected to electrophoresis using diluted 20X MOPS SDS Buffer at 180 V for 60 minutes. The proteins were then visualised using Colloidal Coomassie Stain prepared in-house.

### *2.2.4 In-gel Digestion*

Following de-staining, the gel was washed in MilliQ H2O for 15 minutes. Using a scalpel blade, the lane of protein was excised from the gel and divided into 19 sections. Each section was cut into 1 mm^3^ cubes and placed into a 1.5 mL low-bind tube. A mixture of 100 mM TEAB and 100 % ACN was added to each of the tubes, which were then incubated for 30 minutes at room temperature, with brief vortexing occurring every 5 minutes. An aliquot of 100 % ACN was added to each of the tubes, which were briefly vortexed and incubated until the gel pieces turned opaque. Subsequently, the solution was removed from the tubes and discarded. The samples were reduced by the addition of 10 mM DTT and incubation at 56 °C for 30 minutes. The samples were briefly centrifuged and cooled on ice to room temperature prior to addition of 100 % ACN prior to incubation at room temperature for 10 minutes. Following the removal of all the liquid, 55 mM IAA was added to the tube. The samples were incubated in the dark, at room temperature for 20 minutes before the addition of 100 % ACN to the tubes. Once the gel pieces became opaque, the liquid was removed from the tubes. An aliquot of 40 ng/μL trypsin (prepared with ice-cold 100 mM TEAB) was added to each of the tubes, which were then placed on ice for 2 hours. An aliquot of 100 mM TEAB was overlayed on the gel pieces in each tube before incubating each tube at 37 °C overnight. To halt the digestion, 100 μL of extraction buffer (50 % ACN, 2.5 % FA) was added to each tube, followed by additional incubation at 37 °C for 15 minutes with occasional sonication. The aqueous solution was transferred to a new, labelled tube prior to being dried down using a rotary evaporator. The dried peptides were resuspended in 0.1 % FA (v/v) prior to clean up and desalting using STAGE-Tips.

### *2.2.5 STAGE-Tip*

Stop-and-go-extraction tips (STAGE tips) were utilised to remove any salts and large contaminants from the peptide digests prior to mass spectrometry analysis^1^. The tips were assembled by plugging the end of a 200 μL pipette tip with C18 solid phase extraction membrane. The tips were conditioned and equilibrated by passing 100 % ACN followed by 0.1 % formic acid (FA) through the tip. The samples were then loaded onto the tip, passed through under positive pressure, then washed with 0.1 % FA. The peptides were collected into a MS vial insert by eluting them from the tip using 80 % ACN in 0.1 % FA. The peptides were dried using a rotary vacuum, prior to resuspension in 2 % ACN in 0.1 % FA.

### *2.2.6 iRT Peptides*

Index retention time peptides (iRT peptides) were spiked into the resuspended desalted peptide samples as internal calibrants for MS analysis. These peptides have a known retention time during LC-MS/MS and will allow the data to be used and re-analysed by other researchers.

### *2.3 Protein Processing for Individual Samples*

An acetone precipitation was performed on a 100μL aliquot of the BMSC protein sample to concentrate the proteins and remove large contaminants. The proteins were then resuspended in 0.05M TEAB, a more appropriate buffer for the downstream experiments, and the concentration of protein in each of the samples was quantified using a Bradford Assay as per the manufacturer’s instructions. Filter aided sample preparation (FASP) was utilized to digest the protein samples using trypsin^2^. Briefly, 20 μg of protein of each sample was diluted in Urea buffer (UB: 8 M urea; in 0.1 M Tris / HCl, pH 8.5, 0.025 M DTT) and placed in a Microcon YM-30 centrifugal filter device. The samples were incubated for 1 hour at room temperature then centrifuged at 14,000 × g for 15 minutes at 4 °C (Microfuge® 18 Centrifuge). The proteins were alkylated through the addition of 0.05 M iodoacetamide (IAA) in UB, followed by three washes in UB with centrifugation at 14,000 × g for 15 minutes between washes. The samples were subsequently washed three times in 0.05 M TEAB. Proteins were digested by addition of sequencing grade Trypsin/Lys-C in 0.05 M TEAB, at an enzyme to protein ratio of 1:100, followed by incubation at 37 ºC in a humidified chamber overnight. Finally, the samples were centrifuged, and the peptides collected. After FASP digestion, samples were desalted using the STAGE-Tips, resuspended in ACN and spiked with iRTs as described previously for the pooled sample.

### *2.4 LC-MS/MS*

Liquid chromatography - tandem mass spectrometry (LC-MS/MS) was used to identify and quantify the proteins present in the human BMSC-derived peptide samples using an Eksigent ekspert 400 nanoLC system coupled to a TripleTOF 5600+ mass spectrometer (Sciex). Qualitative mass spectrometry methods were used to generate the data for the protein library while quantitative mass spectrometry methods were used to determine protein abundances in individual samples.

### *2.4.1 Qualitative MS (SWATH-MS)*

The samples were analysed in Data Dependent Acquisition (DDA) mode using a 65-minute gradient. The peptides were separated using a C18 nano-LC resolving column (Eksigent Chrom XP C18 CL-120, 3 μm particle size, 120 Å pore size, 75 μm × 150 mm) with a flow rate of 300 nL / minute across a 45-minute linear gradient of decreasing buffer A (0.1 % FA) from 98 % to 60 % and increasing buffer B (0.1 % FA in 100 % ACN) from 2 % to 40 %, followed by increasing buffer B to 95 % within 3 minutes and sustained for 5 minutes to rinse the stationary phase. Column equilibration was performed with 98 % buffer A for 12 minutes. Subsequently, a peptide identification search, using the generated peptide spectra, was performed using ProteinPilot software and a Swiss-Prot generated, human protein database. The database contained only reviewed and non-redundant proteins. It was concatenated with the common repository of adventitious proteins (cRAP), which was used to account for any contaminants common in mass spectrometry work.

### *2.4.2 Quantitative MS (DDA)*

Using the same chromatographic protocol as for DDA analysis, Data Independent Acquisition (DIA) Mode or Sequential Window Acquisition of All Theoretical Fragment Ion Mass (SWATH-MS) analysis was performed. Similar instrument parameters were used; however, the system was tuned to SWATH-MS acquisition mode instead of DDA mode. SWATH-MS data were imported into the SWATH app within PeakView Software application and ion extraction was performed using the human bMSC protein library to quantify proteins. A false discovery rate (FDR) of 1 % was used to provide robust results.

*2.5 Data analysis*
The spectral data generated was analyzed to understand the state of the MSCs. Differentially expressed proteins that had statistically significant differences (*p* < 0.05) between two groups were identified through Volcano Plot filtering. Hierarchical Clustering was performed to distinguish between control and the treated group. GO and KEGG analysis were used to explore specific functions of the differentially expressed proteins that were identified. A *p*-value was obtained using a hypergeometric test. FDR (corrected *p*-value) <0.05 was set as the cut-off for significant GO terms and KEGG pathways.

**References:**

1. Rappsilber J, Mann M, Ishihama Y. Protocol for Micro-Purification, Enrichment, Pre-Fractionation and Storage of Peptides for Proteomics Using Stagetips. Nat Protoc*.* 2007;2:1896-1906.

2. Wiśniewski JR, Zougman A, Nagaraj N, Mann M. Universal Sample Preparation Method for Proteome Analysis. Nat Methods*.* 2009;6:359-362.
