# Supplementary material for: Effect of fibronectin, FGF-2, and BMP4 in the stemness maintenance of BMSCs and the metabolic and proteomic cues involved
Source: Stem Cell Res Ther. 2021 Mar 6;12:165. doi: 10.1186/s13287-021-02227-7 (PMC7936451; doi:10.1186/s13287-021-02227-7)
Supplement: Supplementary file 5 — Additional file 5: Table S4 and Table S5. [file 13287_2021_2227_MOESM5_ESM.docx]

Table S4 The common KEGG pathways.

| **KEGG pathways** |
| --- |
| Proximal tubule bicarbonate reclamation |
| Arginine and proline metabolism |
| Purine metabolism |
| Basal cell carcinoma |
| Glutathione metabolism |
| Taurine and hypotaurine metabolism |
| Hedgehog signaling pathway |
| Glycine, serine and threonine metabolism |
| Nitrogen metabolism |
| Biosynthesis of unsaturated fatty acids |
| Fatty acid biosynthesis |
| Thiamine metabolism |
| Primary bile acid biosynthesis |
| Pantothenate and CoA biosynthesis |
| Cysteine and methionine metabolism |
| Alanine, aspartate and glutamate metabolism |
| ABC transporters |

(Functional enrichment analysis of 16,126 coding-protein genes was performed by the cluster profiler R package and Venn analysis was performed with the known metabolic pathways identified from the metabolomic characterization).

Table S5 The common KEGG pathways between metabolic and proteomic analysis.

| **KEGG pathways** |
| --- |
| Purine metabolism |
| Alanine, aspartate and glutamate metabolism |
| Glycine, serine and threonine metabolism |
| Cysteine and methionine metabolism |
| Arginine and proline metabolism |
