# Supplementary material for: Effect of fibronectin, FGF-2, and BMP4 in the stemness maintenance of BMSCs and the metabolic and proteomic cues involved
Source: Stem Cell Res Ther. 2021 Mar 6;12:165. doi: 10.1186/s13287-021-02227-7 (PMC7936451; doi:10.1186/s13287-021-02227-7)
Supplement: Supplementary file 6 — Additional file 6: Supplementary Figures. [file 13287_2021_2227_MOESM6_ESM.docx]

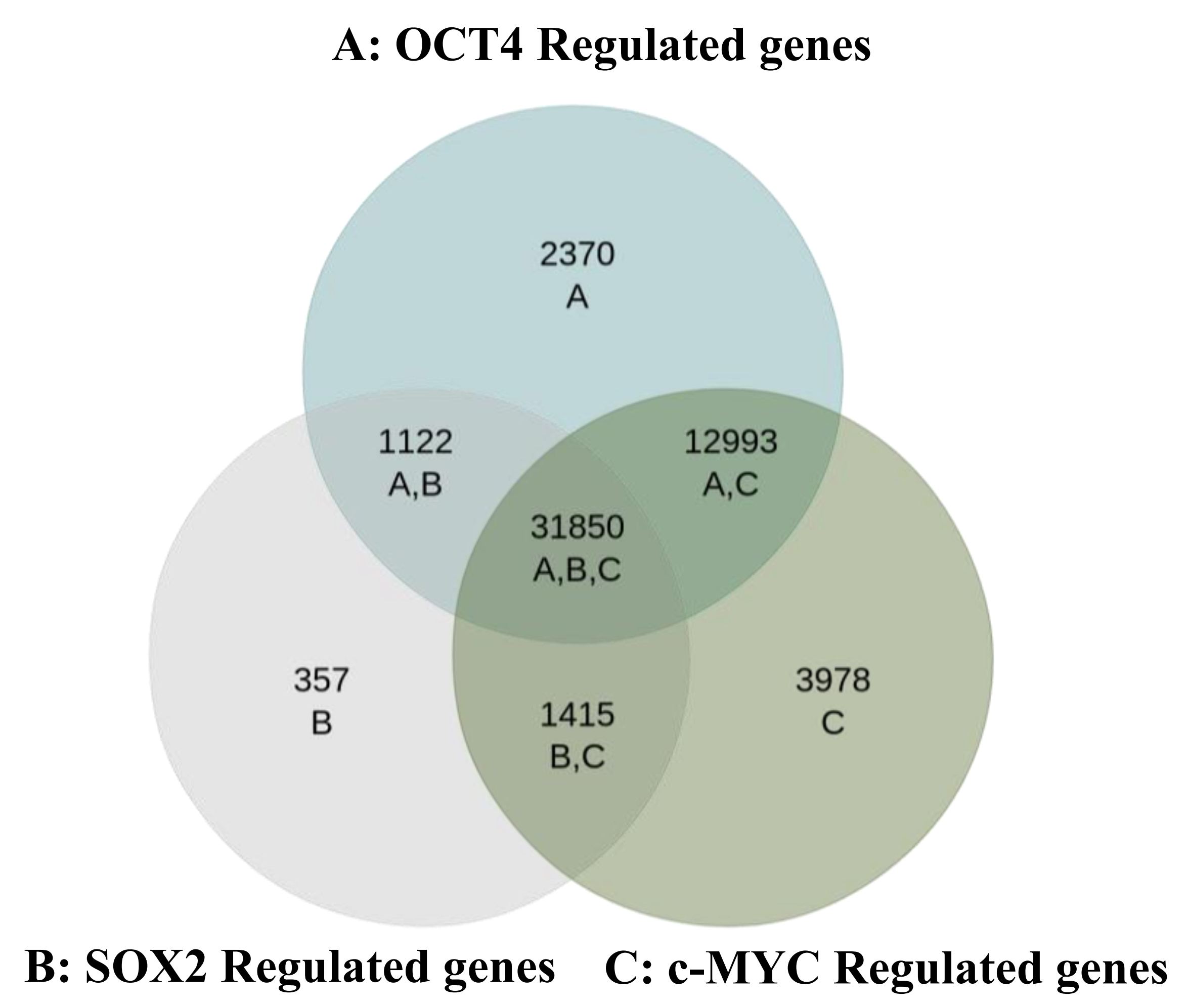
**Supplementary Figures and Figure Legends**

Fig S1 Venn diagram showing 31,850 genes identified across 3 datasets, from which 16,129 commonly identified are coding-protein genes (16,126 to be exact excluding the repeat ones).


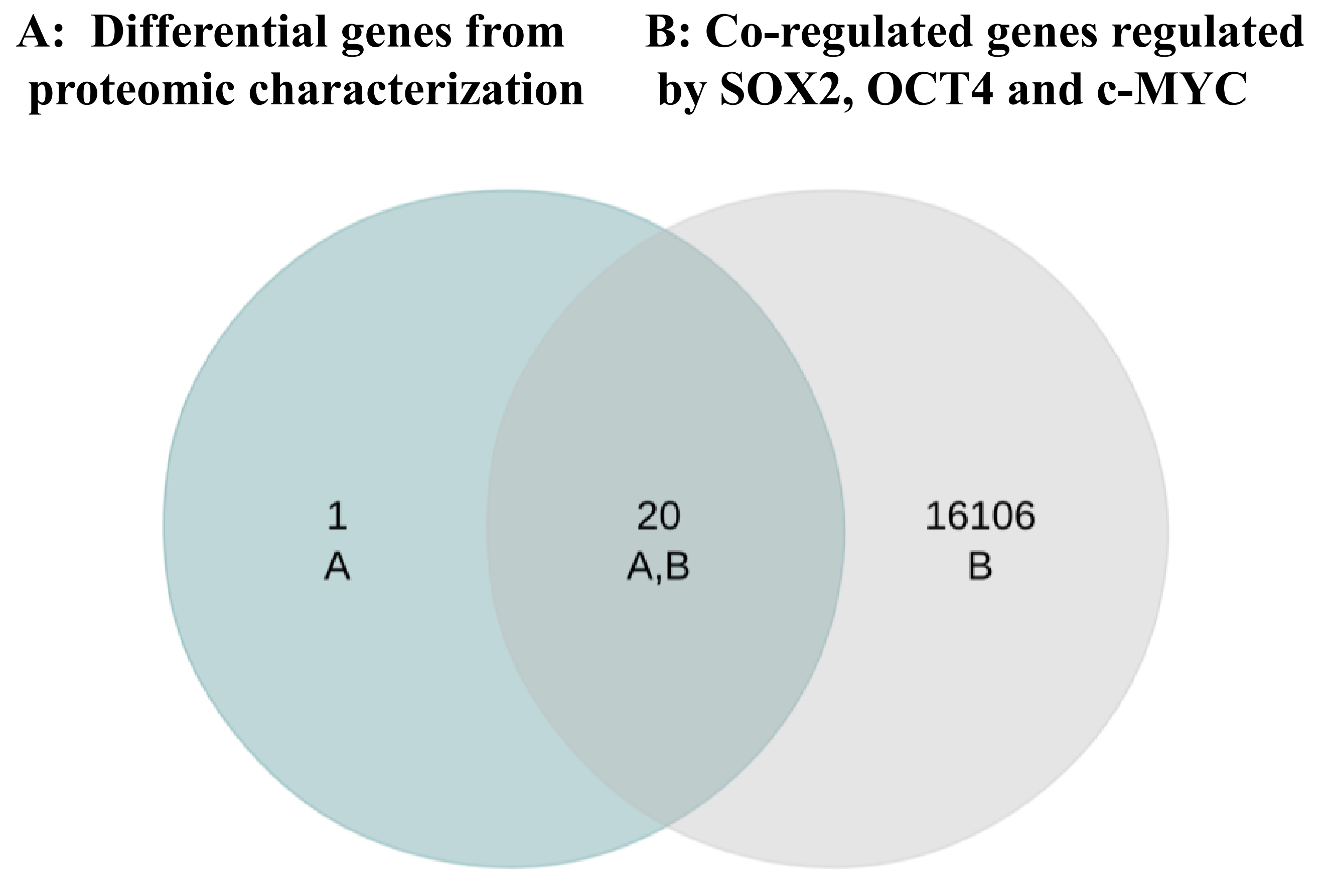
Fig S2 Venn analysis showed 20 co-expressed genes between the 16,126 co-regulated genes and 21 differential genes based on proteomic analysis. The gene outside the intersection was HBB (ENSG ID: ENSG00000244734) which is a potential regulatory gene of SOX2 and c-MYC.

Fig S3
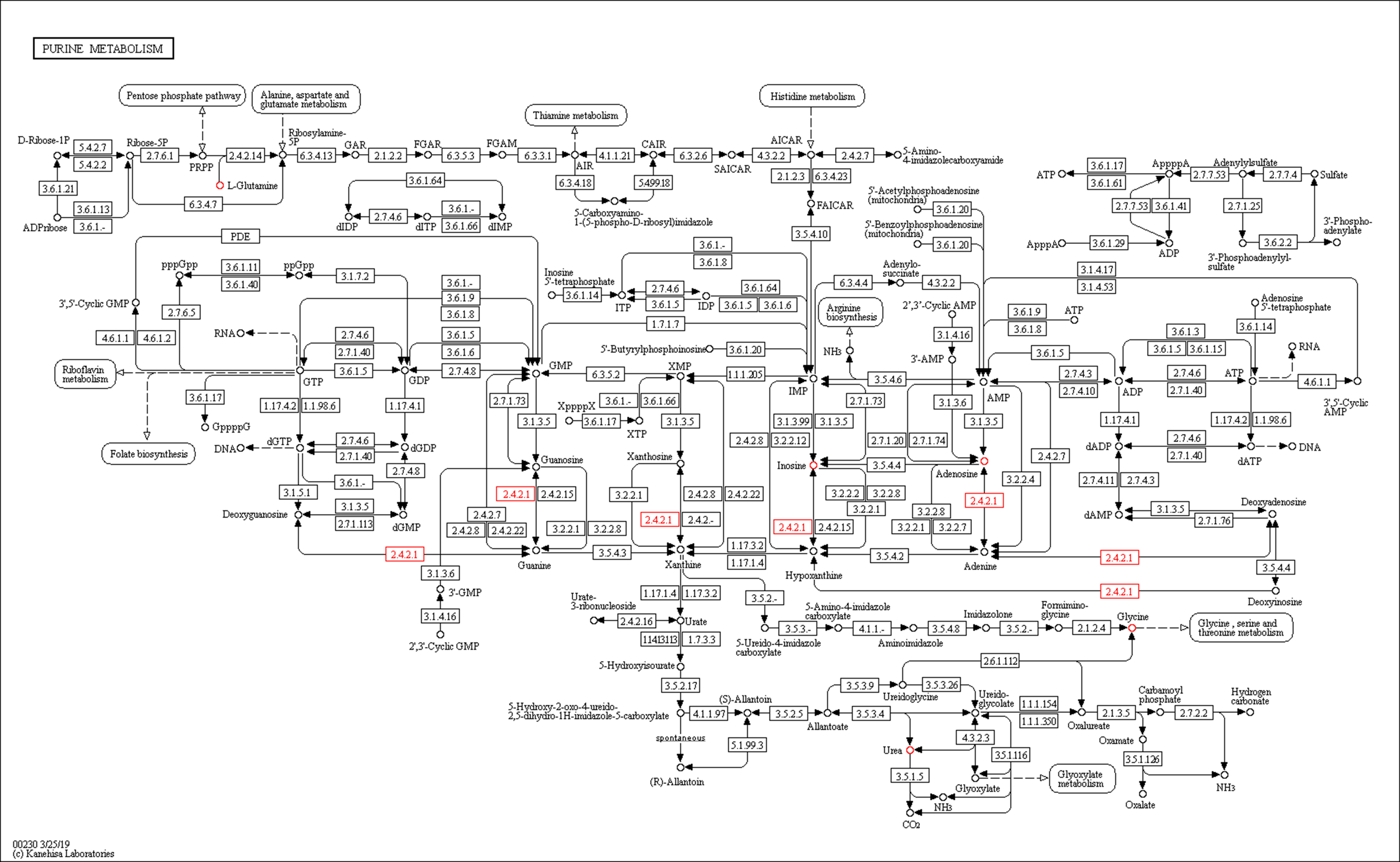
Purine metabolism (KEGG). Purine metabolism from the KEGG database (<http://www.kegg.jp/kegg/pathway/map/map00230.html>), reference image. Protein involved: purine-nucleoside phosphorylase (PNP, EC:2.4.2.1). Metabolites involved: Glycine, L-Glutamate, Urea, L-Proline, Adenosine, Inosine.

Fig S4
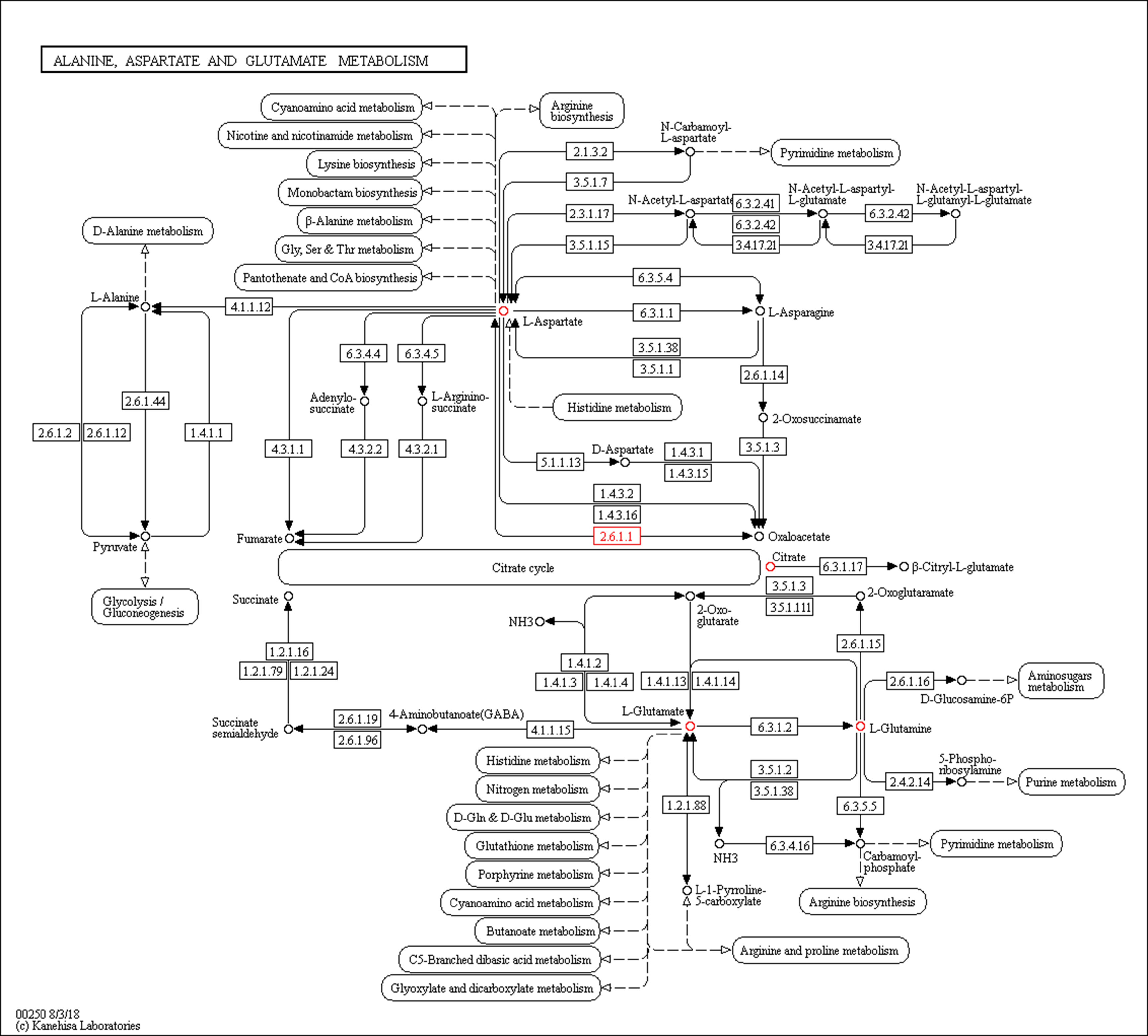
Alanine, aspartate and glutamate metabolism (KEGG). Alanine, aspartate and glutamate metabolism from the KEGG database (https://www.kegg.jp/kegg/pathway/map/map00250.html), reference image. Protein involved: aspartate aminotransferase, cytoplasmic (GOT1, EC:2.6.1.1). Metabolites involved: L-Glutamate, L-Aspartate, L-Glutamine, Citrate.

Fig S5
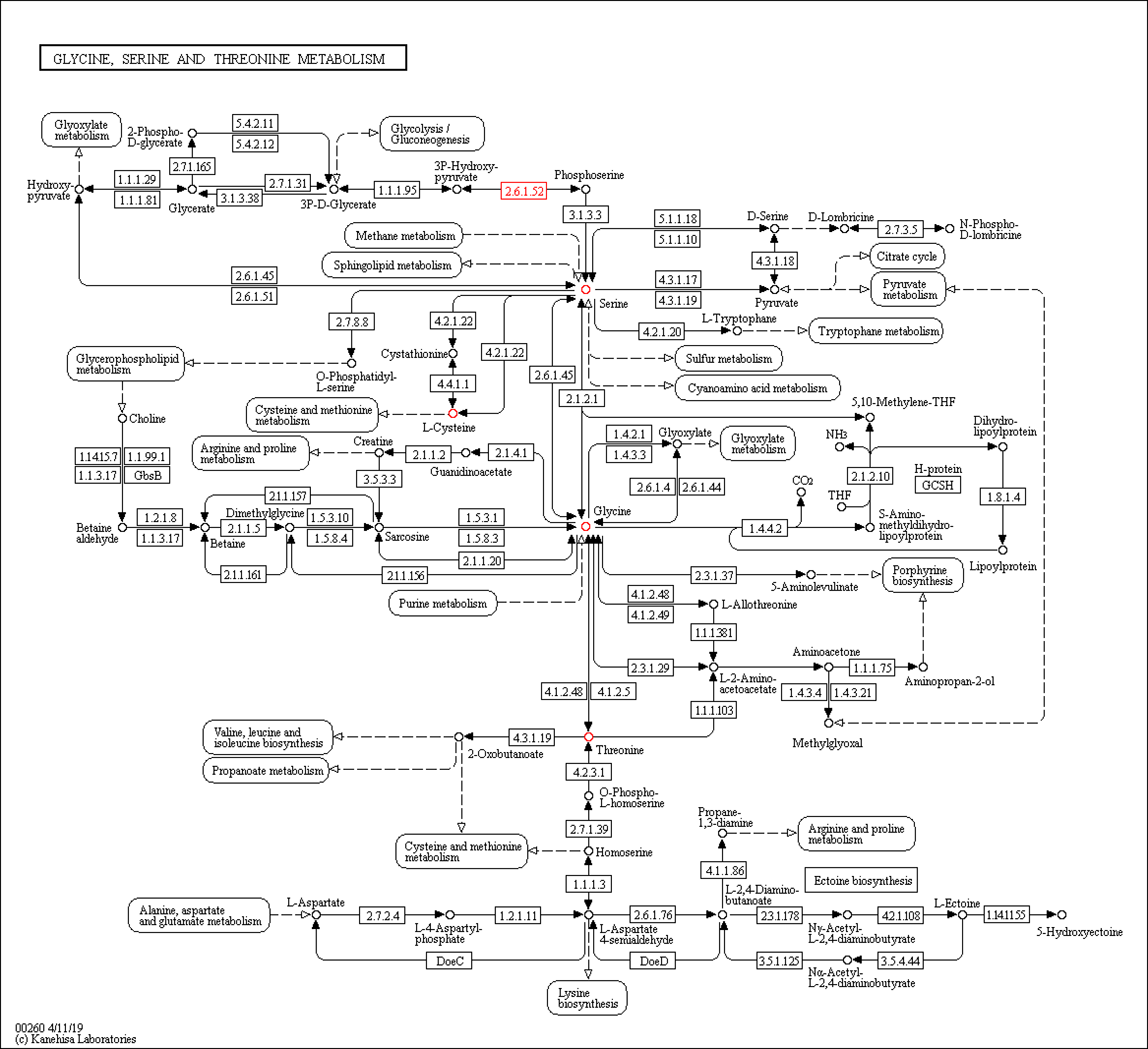
Glycine, serine and threonine metabolism (KEGG). Glycine, serine and threonine metabolism from the KEGG database ([http://www.kegg.jp/kegg/pathway/ map/map00260.html](http://www.kegg.jp/kegg/pathway/%20map/map00260.html)), reference image. Protein involved: phosphoserine aminotransferase (PSAT1, EC:2.6.1.52). Metabolites involved: Glycine, L-Serine, L-Cysteine, L-Threonine.

Fig S6
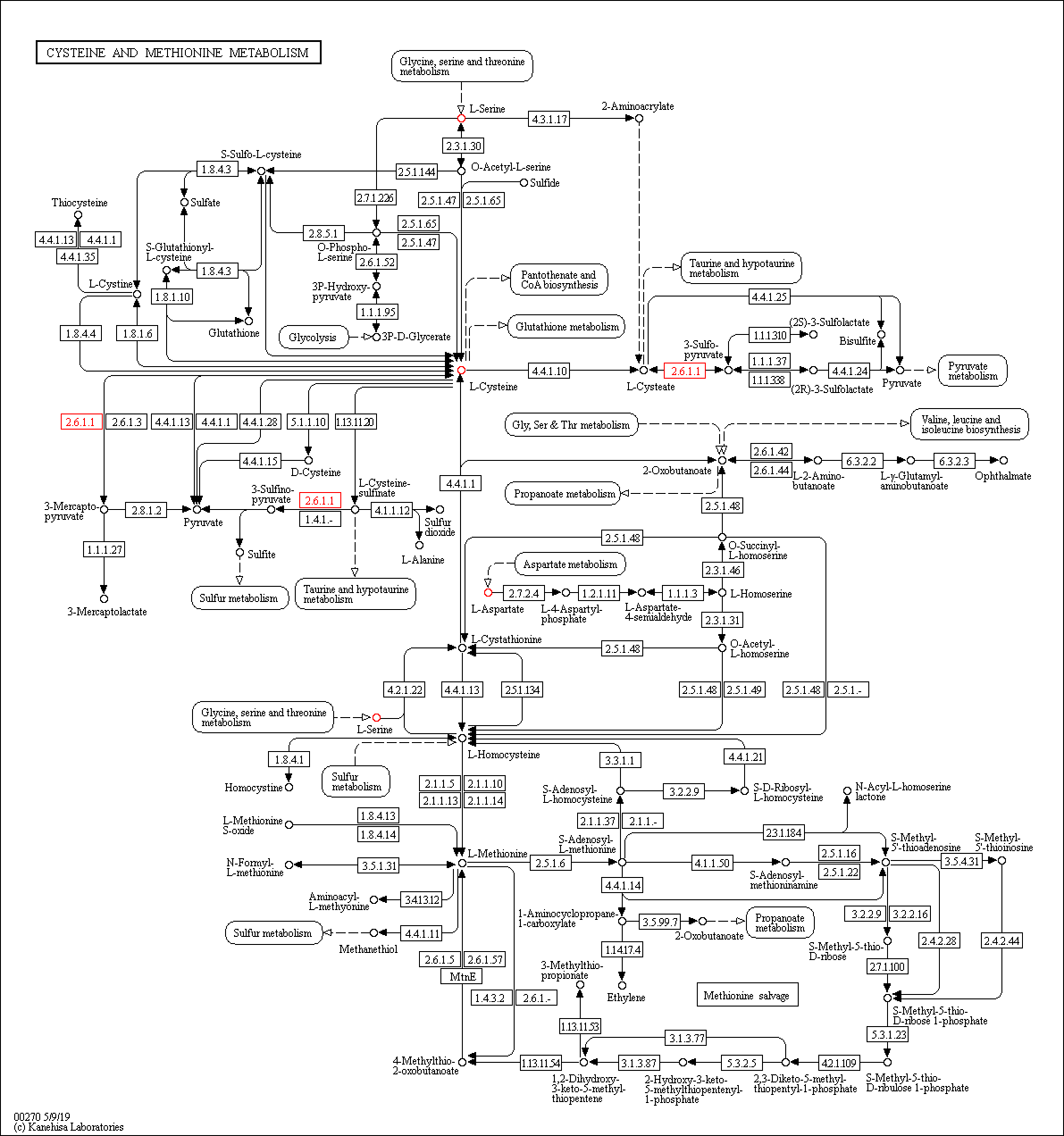
Cysteine and methionine metabolism (KEGG).  Cysteine and methionine metabolism from the KEGG database ([http://www.kegg.jp/kegg/pathway/map/ map00270.html](http://www.kegg.jp/kegg/pathway/map/%20map00270.html)), reference image. Protein involved: aspartate aminotransferase, cytoplasmic (GOT1, EC:2.6.1.1). Metabolites involved: L-Aspartate, L-Serine, L-Cysteine.

Fig S7
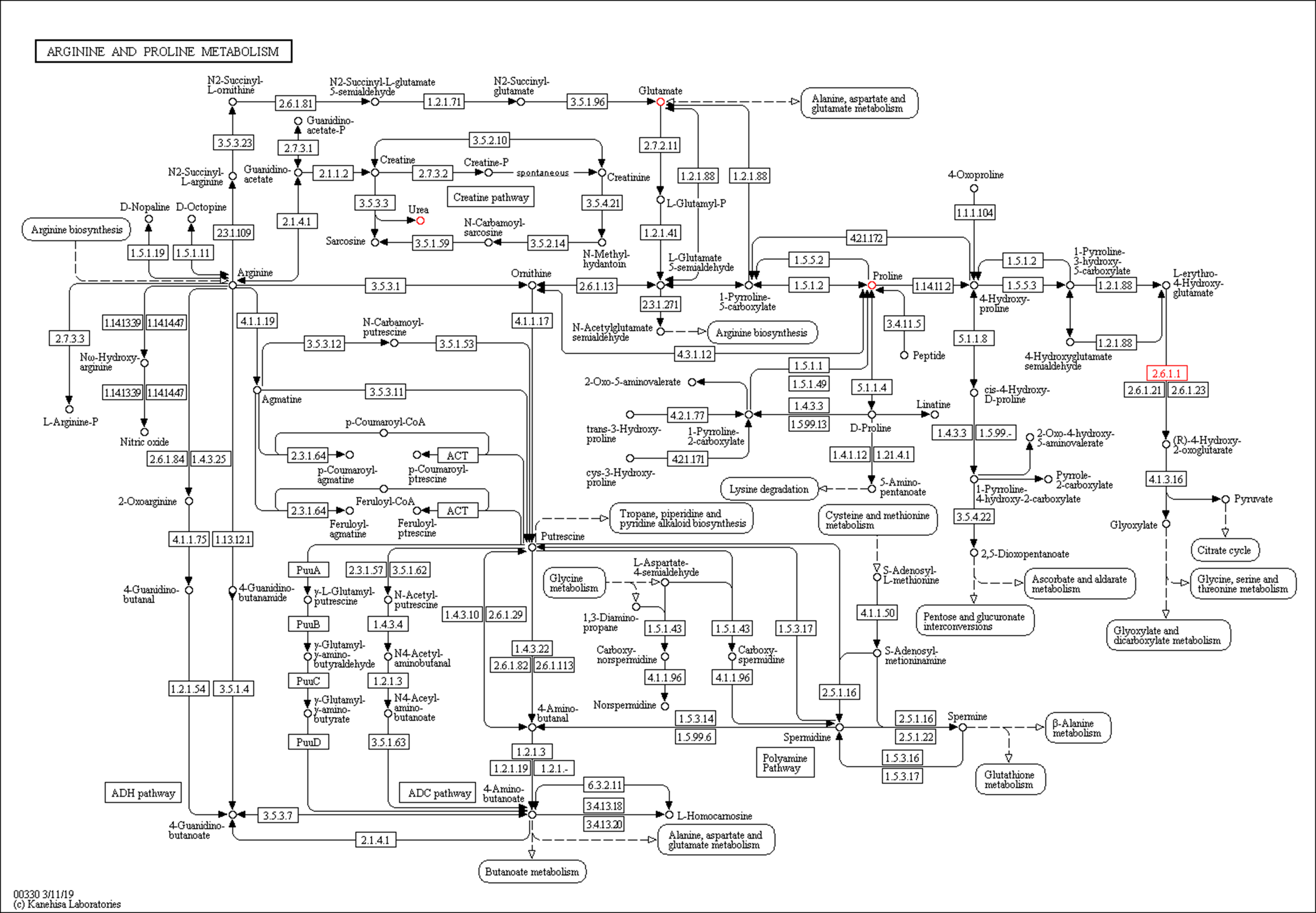
Arginine and proline metabolism (KEGG).  Arginine and proline metabolism from the KEGG database (<http://www.kegg.jp/kegg/pathway/map/map00330.html>), reference image. Protein involved: aspartate aminotransferase, cytoplasmic (GOT1, EC:2.6.1.1). Metabolites involved: L-Glutamate, Urea, L-Proline.
